# Laparoscopic surgery for patients with colorectal cancer produces better short‐term outcomes with similar survival outcomes in elderly patients compared to open surgery

**DOI:** 10.1002/cam4.671

**Published:** 2016-02-29

**Authors:** Soo Yun Moon, Sohee Kim, Soo Young Lee, Eon Chul Han, Sung‐Bum Kang, Seung‐Yong Jeong, Kyu Joo Park, Jae Hwan Oh, the SEoul COlorectal Group (SECOG)

**Affiliations:** ^1^ Center for Colorectal CancerResearch Institute and HospitalNational Cancer CenterGoyangKorea; ^2^ Biometric Research BranchResearch InstituteNational Cancer CenterGoyangKorea; ^3^ Department of SurgerySeoul National University College of MedicineSeoulKorea; ^4^ Department of SurgerySeoul National University Bundang HospitalSeongnamKorea; ^5^ Colorectal Cancer CenterSeoul National University Cancer HospitalSeoulKorea; ^6^ Cancer Research InstituteSeoul National UniversitySeoulKorea

**Keywords:** Colorectal cancer, elderly patients, laparoscopic surgery, surgical outcomes

## Abstract

The number of operations on elderly colorectal cancer (CRC) patients has increased with the aging of the population. The aim of this study was to evaluate surgical outcomes in elderly patients who underwent laparoscopic or open surgery for CRC. We analyzed the data of 280 patients aged 80 or over who underwent surgery for CRC between January 2001 and December 2010. Seventy‐one pairs were selected after propensity score matching for laparoscopic or open surgery. Operative time, return to normal bowel function, length of hospital stay, postoperative complications, overall survival (OS), recurrence‐free survival (RFS), and prognostic factors affecting survival were investigated. In matched cohorts, operative time in the laparoscopic group was longer than in the open group (*P* < 0.001). In the laparoscopic group, time to flatus passage (*P* < 0.001) and length of postoperative hospital stay (*P* = 0.037) were shorter than in the open group. The rate of operation‐related morbidity was higher in the open group (*P* = 0.019). There was no difference in OS and RFS between two groups. This study suggests that laparoscopic surgery for CRC in elderly patients may be safe and feasible, with better short‐term outcomes. OS and RFS, however, were not different in both groups.

## Introduction

Improvements in health care and advances in medicine have led to an increase in life expectancy 1. The increasingly elderly population is associated with a high incidence of colorectal cancer (CRC) requiring surgical intervention. Because of the reduction in physiological reserve, elderly patients may not tolerate surgery 2. In addition, the elderly are more likely to have comorbid conditions, in particular pulmonary and cardiovascular disease, which are mainly responsible for the higher morbidity and mortality rate 3.

The clinical benefits of laparoscopic colorectal surgery, including reduced postoperative pain, early recovery, and shorter hospitalization have been increasingly appreciated 1, 3, 4, 5. The advantages of laparoscopic surgery may be more beneficial in elderly patients with comorbid conditions 6.

However, most studies of laparoscopic surgery in elderly patients were limited by small numbers and their retrospective nature 7, 8. Moreover, very few studies reported long‐term outcomes 9, 10.

Therefore, we conducted a multicenter, propensity score‐matched analysis comparing laparoscopic and open colorectal surgery in elderly patients aged at least 80. The aim of this study was to evaluate surgical outcomes of laparoscopic surgery compared with those of open surgery for elderly patients with CRC.

## Materials and Methods

### Patients

We analyzed the short‐term and survival outcomes of patients aged 80 or over who underwent surgery for CRC at three tertiary referral hospitals in South Korea (the National Cancer Center, the Seoul National University Hospital, and the Seoul National University Bundang Hospital) between January 2001 and December 2010. Exclusion criteria included a synchronous CRC or distant metastasis, medical history of other primary malignancy, combined operations for other disease, emergency operation, and patients who received preoperative chemotherapy, radiotherapy, or chemoradiotherapy. The choice of type of surgery was determined by the individual surgeon's preference. Of 280 patients included in this study, 85 underwent laparoscopic resection and 195 underwent open resection. Among the 280 patients, 142 were matched using propensity scoring. Data were collected in a prospectively maintained database that was supplemented by retrospective chart review. This study was approved by the institutional review board of each participating hospital (NCC2014‐0009, H‐1401‐084‐550, B‐1401‐2301‐112).

Patient characteristics assessed included age, sex, body mass index (BMI), American Society of Anesthesiologists (ASA) score, comorbidity, tumor stage, tumor location, and preoperative carcinoembryonic antigen (CEA) level. Tumor stage was based on final pathologic assessment. Tumor location was divided into three areas: right colon, left colon, and rectum. Right colon was defined as cecum, ascending colon, hepatic flexure colon, and transverse colon; left colon was defined as splenic flexure colon, descending colon, and sigmoid colon. Perioperative factors were compared, including operative time, estimated blood loss (EBL), type of resection, conversion rate, recovery results, postoperative morbidity, mortality, and pathologic data. Conversion to open operation was defined as an abdominal incision larger than necessary for specimen retrieval 11. Tumor grade was divided into two groups. Low grade included well‐differentiated and moderately differentiated adenocarcinoma. High grade included poorly differentiated and mucinous adenocarcinoma, and signet ring cell carcinoma. Postoperative management was the same for laparoscopic and open groups. Diet was resumed after flatus had passed. Postoperative complications were monitored for 30 days, and were defined as any event requiring specific medical or surgical treatment. Complications were categorized as wound infection, ileus, voiding difficulty, anastomotic leakage, intra‐abdominal bleeding, pneumonia, or other complications. Other complications included poor oral intake without evidence of mechanical ileus, chylous ascites, delirium, atrial fibrillation, acute renal failure due to a high‐output stoma, and vocal fold atrophy. Mortality was defined as death within 30 days after surgery. Factors contributing to survival underwent univariable and multivariable analysis.

### Statistical analysis

Differences in baseline characteristics between the two groups (open surgery vs. laparoscopic surgery) were examined using chi‐square, Fisher's exact, and Wilcoxon rank‐sum tests as appropriate. To adjust for the differences in baseline characteristic between the two groups, a propensity score was developed using the logistic regression model. Variables used in the propensity model were age, sex, BMI, ASA score, comorbidity, pathological tumor–node–metastasis (TNM) stage, tumor location, and preoperative CEA level. Subsequently, a one‐to‐one match between two groups was obtained using nearest neighbor matching with a caliper method 12.

The Kaplan–Meier method, the log‐rank test, and the Cox regression model were used for survival analyses. Only factors with *P*‐values <0.10 in the univariable analysis were subsequently evaluated in a Cox proportional hazards regression analysis using backwards stepwise selection with a 0.10 significance level for overall survival (OS) and recurrence‐free survival (RFS) as endpoints. A *P*‐value less than 0.05 was considered as statistically significant and confidence intervals (CI) were at 95% level. Statistical analyses were performed using STATA^®^ version 13 (StataCorp LP, TX).

## Results

### Baseline characteristics

Of 280 patients included in this study, 30% (85/280) underwent laparoscopic resection and 70% (195/280) underwent open resection. After performing propensity score matching for the entire study population, 71 matched pairs of patients were selected. Baseline characteristics of the pre‐ and postmatching groups are outlined in Table 1. Before matching, there were differences between the two groups. Tumor stage was higher in the open group compared with the laparoscopic group (*P* = 0.002). Tumor location in the right colon and rectum was more frequent in the open than the laparoscopic group (*P* = 0.007). After matching, the two groups were well balanced in terms of age, sex, BMI, ASA score, comorbidity, stage of the tumor, tumor location, and preoperative CEA level. The median age was 82 years old in both groups (*P* = 0.711).

**Table 1:** Patient characteristics before and after matching by propensity score

	Total cohort			Matched cohort		
	LAP (*n* = 85)	OP (*n* = 195)	*P*	LAP (*n* = 71)	OP (*n* = 71)	*P*
Age (years)						
Median (IQR)	82.0 (80–84)	82.0 (81–85)	0.055	82.0 (81–84)	82.0 (81–84)	0.711
Sex			0.552			1.000
Male	46 (54.1)	98 (50.3)		40 (56.3)	40 (56.3)	
Female	39 (45.9)	97 (49.7)		31 (43.7)	31 (43.7)	
BMI (kg/m^2^)						
Median (IQR)	22.2 (20.6–24.1)	21.6 (19.5–23.6)	0.093	21.9 (20.4–24.2)	22.0 (20.6–24.5)	0.901
ASA score			0.882			0.396
1	13 (15.3)	35 (17.9)		12 (16.9)	16 (22.5)	
2	56 (65.9)	120 (61.5)		45 (63.4)	37 (52.1)	
3	16 (18.8)	38 (19.5)		14 (19.7)	18 (25.4)	
4	0 (0.0)	2 (1.0)				
Comorbidity	55 (64.7)	108 (55.4)	0.146	44 (62.0)	40 (56.3)	0.495
Hypertension	49 (57.6)	90 (46.2)		40 (56.3)	34 (47.9)	
Diabetes mellitus	10 (11.8)	26 (13.3)		10 (14.1)	10 (14.1)	
Cardiovascular disease	8 (9.4)	10 (5.1)		4 (5.6)	3 (4.2)	
Cerebrovascular disease	4 (4.7)	7 (3.6)		2 (2.8)	5 (7.0)	
Pulmonary disease	3 (3.5)	4 (2.1)		2 (2.8)	0 (0.0)	
TNM Stage			0.002			0.978
I	26 (30.6)	25 (12.8)		19 (26.8)	18 (25.4)	
II	28 (32.9)	77 (39.5)		24 (33.8)	24 (33.8)	
III	31 (36.5)	93 (47.7)		28 (39.4)	29 (40.8)	
Tumor location			0.007			0.913
Right colon	19 (22.4)	61 (31.3)		17 (23.9)	15 (21.1)	
Left colon	44 (51.8)	62 (31.8)		34 (47.9)	36 (50.7)	
Rectum	22 (25.9)	72 (36.9)		20 (28.2)	20 (28.2)	
Preoperative CEA (ng/mL)			0.179			0.579
≤5	59 (75.6)	117 (67.2)		52 (73.2)	49 (69.0)	
>5	19 (24.4)	57 (32.8)		19 (26.8)	22 (31.0)	

Data are presented as *n* (%) unless otherwise indicated.

LAP, laparoscopic surgery; OP, open surgery; IQR, interquartile range; BMI, body mass index; ASA, American Society of Anesthesiologists; TNM, tumor node metastasis; CEA, carcinoembryonic antigen.

### Short‐term outcomes

Operative outcomes in matched cohorts are presented in Table 2. Operative time in the laparoscopic group was longer than in the open group (182.0 vs. 130.0 min, *P* < 0.001). EBL did not differ between the two groups (*P* = 0.637). Cases with more than 12 harvested lymph nodes (LNs) were more frequent in the laparoscopic than the open group (94.4 vs. 77.5%, *P* = 0.004). Time to first flatus (3.0 vs. 4.0 days, *P* < 0.001) and time to resume soft diet (5.0 vs. 6.0 days, *P* = 0.004) were shorter in the laparoscopic than the open group. The length of postoperative hospital stay (9.0 vs. 10.0 days, *P* = 0.037) was also shorter in the laparoscopic than the open group.

**Table 2:** Perioperative outcomes in matched cohorts of laparoscopic and open surgery

	LAP (*n* = 71)	OP (*n* = 71)	*P*
Type of resection			0.385
Right hemicolectomy	17 (23.9)	15 (21.1)	
Left hemicolectomy	3 (4.2)	4 (5.6)	
Anterior resection	24 (33.8)	23 (32.4)	
Low anterior resection	24 (33.8)	19 (26.8)	
Miles' operation	2 (2.8)	2 (2.8)	
Hartmann's operation	1 (1.4)	4 (5.6)	
Subtotal colectomy		4 (5.6)	
Operative time (min)			
Median (IQR)	182.0 (154–210)	130.0 (80–185)	<0.001
EBL (mL)			
Median (IQR)	100.0 (50–200)	100.0 (50–300)	0.637
Harvested LN			0.004
<12	4 (5.6)	16 (22.5)	
≥12	67 (94.4)	55 (77.5)	
Tumor grade			0.573
Low	65 (91.5)	63 (88.7)	
High	6 (8.5)	8 (11.3)	
Venous invasion	19 (26.8)	21 (29.6)	0.709
Angiolymphatic Invasion	35 (49.3)	20 (28.2)	0.010
Perineural invasion	16 (22.5)	20 (28.2)	0.440
Flatus passage (days)			
Median (IQR)	3.0 (2–4)	4.0 (4–6)	<0.001
First soft diet (days)			
Median (IQR)	5.0 (4–6)	6.0 (5–7)	0.004
Hospital stay (days)			
Median (IQR)	9.0 (7–11)	10.0 (8–13)	0.037

Data are presented as *n* (%) unless otherwise indicated.

LAP, laparoscopic surgery; OP, open surgery; IQR, interquartile range; EBL, estimated blood loss; LN, lymph node.

Postoperative complications occurred in 22.5% and 40.8% in the laparoscopic and open group, respectively (Table 3, *P* = 0.019). In the open group, the most common morbidity was postoperative ileus in 11 patients (15.5%), followed by wound infection in nine (12.7%), and urinary retention in nine (12.7%). In the laparoscopic group, the most common morbidity was urinary retention in nine patients (12.7%), followed by wound infection in five (7.0%). Among nine patients experienced urinary retention in the laparoscopic group, five patients had colon cancer and four patients had rectal cancer (*P* = 0.245). The incidence of postoperative complications was not significantly different between Hartmann's operation group and the other surgery group (*P* = 0.684). Among 15 patients with protective ileostomy or colostomy, seven patients were experienced postoperative complications (two patients in wound infection, one patient in ileus, three patients in voiding difficulty, one patient in atrial fibrillation, one patient in vocal fold atrophy), and diversion was not statistically associated with complication rate (*P* = 0.195). There was no 30‐day postoperative mortality in matched groups. In the total cohort, one patient from the open group died within 30 days of surgery because of pulmonary thromboembolism. Conversion to open surgery was necessary in five laparoscopic group patients; there was a pelvic abscess due to sealed‐off perforation of the sigmoid colon in one patient, too narrow a pelvic cavity to perform dissection in two patients, ureter adhesion to tumor in one patient, and superior mesenteric vein injury in one patient.

**Table 3:** Postoperative complications in matched cohorts of laparoscopic and open surgery

	LAP (*n* = 71)	OP (*n* = 71)	*P*
Postoperative mortality	0 (0)	0 (0)	
Postoperative morbidity	16 (22.5)	29 (40.8)	0.019
Wound infection	5 (7.0)	9 (12.7)	0.260
Ileus	3 (4.2)	11 (15.5)	0.024
Urinary retention	9 (12.7)	9 (12.7)	1.000
Anastomosis leakage	0 (0.0)	0 (0.0)	1.000
Intra‐abdominal bleeding	1 (1.4)	1 (1.4)	1.000
Pneumonia	1 (1.4)	0 (0.0)	1.000
Other complication	4 (5.6)	2 (2.8)	0.681

Data are presented as *n* (%) unless otherwise indicated.

LAP, laparoscopic surgery; OP, open surgery.

### Prognostic factors affecting OS and RFS

**Figure 1:**
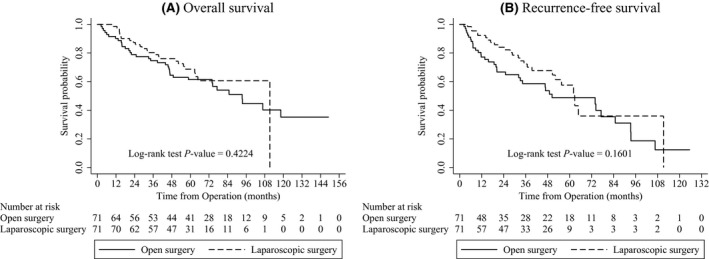
Survival curve in matched cohorts of laparoscopic and open surgery.

The median follow‐up period was 68.3 months (IQR [interquartile range] 57.4–88.7 months; laparoscopic group 61.9 months, open group 72.2 months). In the matched cohorts, 60 of 142 patients died and 61 of 142 had local or distant recurrence during the follow‐up period. In matched cohorts, OS and RFS were similar in both groups (Fig. 1). In subgroup analysis of the patients with colon and rectal cancers separately, OS and RFS were not significantly different between the laparoscopic and open groups both in patients with colon cancer and rectal cancer (colon cancer: Fig. S1, *P* = 0.776 for OS, *P* = 0.335 for RFS; rectal cancer: Fig. S2, *P* = 0.32 for OS, *P* = 0.349 for RFS). Factors affecting survival are presented in Tables 4 and 5. Perineural invasion (*P* = 0.020) was significantly associated with poorer OS in univariable analysis (Table S1). Males (*P* = 0.055) and patients who received Hartmann's operation (*P* = 0.064) were marginally associated with poorer OS in univariable analysis. In multivariable analysis with these variables, perineural invasion (hazard ratio [HR], 1.79; 95% CI, 1.02–3.15; *P* = 0.043) was the only significant prognostic factor affecting OS.

**Table 4:** Multivariable analysis for overall survival in matched cohorts of laparoscopic and open surgery

	HR	95% CI	*P*
Sex			
Male	1.00		
Female	0.62	0.36–1.07	0.085
Perineural invasion			
No	1.00		
Yes	1.79	1.02–3.15	0.043
Type of surgery			
OP	1.00		
LAP	0.82	0.48–1.38	0.457

LAP, laparoscopic surgery; OP, open surgery; HR, hazard ratio; CI, confidence interval.

**Table 5:** Multivariable analysis for recurrence‐free survival in matched cohorts of laparoscopic and open surgery

	HR	95% CI	*P*
Preoperative CEA (ng/mL)			
≤5	1.00		
>5	1.81	1.04–3.14	0.035
Perineural invasion			
No	1.00		
Yes	1.90	1.08–3.32	0.025
Type of surgery			
OP	1.00		
LAP	0.67	0.40–1.12	0.125

LAP, laparoscopic surgery; OP, open surgery; HR, hazard ratio; CI, confidence interval; CEA, carcinoembryonic antigen.

High preoperative CEA level (*P* = 0.027) and perineural invasion (*P* = 0.014) were associated with poorer RFS in univariable analysis (Table S2). Angiolymphatic invasion (*P* = 0.092) was marginally associated with poorer RFS. Among these variables, prognostic factors affecting RFS were high preoperative CEA level (HR, 1.81; 95% CI, 1.04–3.14; *P* = 0.035) and perineural invasion (HR, 1.90; 95% CI, 1.08–3.32; *P* = 0.025). Surgical method (either laparoscopic surgery or open surgery) was not associated with OS and RFS in multivariable analysis. Also, in subgroup analysis dividing into the patients with colon and rectal cancers, surgical method was not associated with OS and RFS (Tables S3, S4).

## Discussion

This multicenter, propensity score matched study compared the short‐term and survival outcomes of elderly patients who underwent laparoscopic or open resection for CRC. Our results suggested that laparoscopic colorectal surgery in elderly patients achieved better short‐term outcomes and similar OS and RFS compared to open surgery.

Several studies demonstrated that laparoscopic surgery for colorectal disease in elderly patients has short‐term benefits in terms of earlier recovery of bowel function and shorter length of hospital stay 3, 5. A case‐matched study by Vignali et al. compared outcomes for 61 octogenarian patients after laparoscopic surgery with those for the same number of patients undergoing open surgery, and reported that a faster recovery of bowel function (4.8 vs. 5.9 days, *P* = 0.003) and a shorter hospital stay (9.8 vs. 12.9 days, *P* = 0.001) were achieved in the laparoscopic group 3. A randomized control study of elderly patients aged 75 years or older also demonstrated that length of hospitalization was statistically shorter for laparoscopic (10.0 days) than open surgery (13.0 days, *P* = 0.026) for colon cancer 5. The shorter length of hospital stay observed in the laparoscopic group could be ascribed to the earlier recovery of bowel function and to the better return to full independence 3. Consistent with previous reports, our study showed earlier recovery of bowel function and shorter length of hospital stay in the laparoscopic group.

Elderly patients have a higher frequency of complications and mortality rate after surgery than younger patients 13, 14. In many reports, however, old age itself is not an independent prognostic factor for colorectal surgery 6, 7. In a systematic review of the correlation between age and outcomes of CRC surgery, selected elderly patients benefited from surgery, since a large proportion survived for 2 or more years, irrespective of their age 15.

However, there is controversy about whether laparoscopic surgery has fewer complications than open surgery. Stocchi et al. reported a lower incidence of postoperative complications in patients aged more than 75 years who were operated upon laparoscopically (14.3%) compared with those who underwent open surgery (33.3%) in a matched control study of 42 patients each (*P* = 0.04) 16. A multicenter‐matched case–control study by Hinoi et al. also reported that the complication rates were 24.9% and 36.3% for laparoscopic surgery and open surgery, respectively, in colon cancer patients older than 80 years 9. In contrast, several studies reported similar rates of postoperative complications between laparoscopic surgery and open surgery in elderly patients 3, 4. In this study, laparoscopic surgery was associated with less frequent morbidity than open surgery. In particular, there was less postoperative ileus in the laparoscopic group. The incidence of adhesive ileus after laparoscopic colorectal surgery was reported to be very low (2–4%) 17, 18. A study comparing laparoscopic colorectal to open surgery showed that laparoscopic surgery is associated with reduced rates of adhesive intestinal obstruction 19.

In our study, we reported the relatively high rate of wound infection (7% in the laparoscopic group, 12.7% in the open group), but the incidence of wound infection was reported variously in previous studies. A case‐matched study by Vignali et al. compared outcomes for octogenarian patients reported that the incidence of wound infection were 8.1% in the laparoscopic group and 14.7% in the open group 3. Frasson et al. also reported similar infection rate as 6.7% in the laparoscopic group and 16.1% in the open group for patients aged over 70 years 6.

All patients in this study underwent radical surgery with LN dissection. Adequate resection of LNs in surgery of CRC is an important factor in tumor staging. Several factors have been shown to have a positive correlation with the number of LNs harvested, such as patient age <60 years, right‐sided tumor location, advanced tumor stage, longer length of resected bowel segment, and expertise of the pathologist and surgeon 20, 21, 22. In this study, the median numbers of LNs harvested were 26 in the laparoscopic and 20 in the open group. The proportion of patients in whom more than 12 LNs were harvested was higher in the laparoscopic than the open group. More accurate LN dissection in laparoscopic surgery became possible because laparoscopy provided improved visualization 23. However, more LN dissection in laparoscopic surgery did not result in improvement of OS and RFS in elderly patients.

Several randomized controlled trials have confirmed that the long‐term surgical outcomes of laparoscopic surgery are similar to those of open surgery for colon or rectal cancer, in terms of local recurrence or overall survival 24, 25, 26. Few reports, however, provided information related to long‐term survival outcomes for elderly patients who underwent laparoscopic‐assisted colorectal resection 9, 10. A large‐scale multicenter‐matched case–control study from Japan comparing outcomes for patients aged 80 or over reported that 3‐year OS, disease‐free survival (DFS), and cancer‐specific survival did not differ between the laparoscopic and open group 9. Consistent with previous studies, OS and RFS did not differ in both groups in our study.

After a median 68.3 months of follow‐up, our study showed that 3‐year OS rates following laparoscopic and open resection are 80.3% and 74.7%, respectively, for elderly patients. These results are comparable to other studies (Medical Research Council Conventional vs. Laparoscopic‐Assisted Surgery in Colorectal Cancer [MRC‐CLASSICC] and COlon carcinoma Laparoscopic or Open Resection [COLOR]) for the general population 25, 27.

We evaluated several possible prognostic factors that may influence survival in elderly patients with CRC, including age, underlying disease, TNM stage, grade of tumor differentiation, preoperative CEA level, lymphovascular invasion, perineural invasion, and postoperative complications 28, 29, 30, 31, 32, 33, 34, 35.

Our study showed perineural invasion was an independent risk factor for OS and RFS in elderly patients. In a meta‐analysis, perineural invasion has an unfavorable impact on OS and DFS in CRC patients. Further subgroup analysis revealed that the significance of the association between perineural invasion and worse prognosis is not affected by many factors, including TNM stage and tumor site 30. Other studies also demonstrated that perineural invasion is a stage‐independent prognostic factor 33, 34. Pre‐existing structures used as a potential route of metastasis include veins, lymphatic channels, and the nervous system; the nervous system was demonstrated to be most closely associated with postoperative recurrence in CRC. A higher grade of perineural invasion is associated with not only locoregional recurrence but also recurrence in distant organs such as the liver, lung, and peritoneum 35.

In this study, elevated preoperative CEA levels have an adverse impact on RFS. High preoperative CEA level suggests advanced disease 36. Wiratkapun et al. reported that the cumulative DFS of patients with a preoperative CEA level within the normal range was significantly better than that of those whose CEA was higher than normal 32.

In our study, TNM stage was not an independent risk factor for OS and RFS. Increased HR with advancing stage in univariable analysis (Tables S1, S2) did not reach statistical significance, which might reflect a small sample size or deaths unrelated to CRC.

This study has limitations. First, it is a retrospective study with inherent selection bias. To minimize this, patients undergoing laparoscopic surgery were carefully matched to patients undergoing open surgery using propensity scoring. Second, we did not analyze cancer‐specific survival. Since elderly patients are at higher risk of dying from other disorders, it is important to know death rates associated with CRC. Third, the statistical power is insufficient because sample size is small after matching. Finally, despite the postoperative quality of life (QOL) including degree of functional loss is one of major issues in the geriatric surgery, QOL data could not be assessed due to the lack of records in this retrospective study.

This study showed that laparoscopic colorectal surgery in elderly patients achieved better results than open surgery in terms of bowel function recovery, length of hospital stay, and postoperative complications. OS and RFS following laparoscopic and open colorectal resection were similar in the elderly population. These findings suggest that laparoscopic colorectal surgery in elderly patients is safe and feasible, and should be considered as a treatment option.

## Conflict of Interest

The authors claim no conflicts of interest.

## Supporting information


**Figure S1.** Survival curve in colon cancer patients according to type of surgery.

**Figure S2.** Survival curve in rectal cancer patients according to type of surgery.

**Table S1.** Factors associated with overall survival in matched cohorts on univariable and multivariable analysis.

**Table S2.** Factors associated with recurrence‐ free survival in matched cohorts on univariable and multivariable analysis.

**Table S3.** Multivariable analysis for overall survival in matched cohorts of laparoscopic and open surgery: subgroup analysis of patients with colon and rectal cancers.

**Table S4.** Multivariable analysis for recurrence‐free survival in matched cohorts of laparoscopic and open surgery: subgroup analysis of patients with colon and rectal cancers.
