# Supplementary figures and images for: Laparoscopic surgery for patients with colorectal cancer produces better short‐term outcomes with similar survival outcomes in elderly patients compared to open surgery
Source: Cancer Med. 2016 Feb 29;5(6):1047–54. doi: 10.1002/cam4.671 (PMC4924362; doi:10.1002/cam4.671)

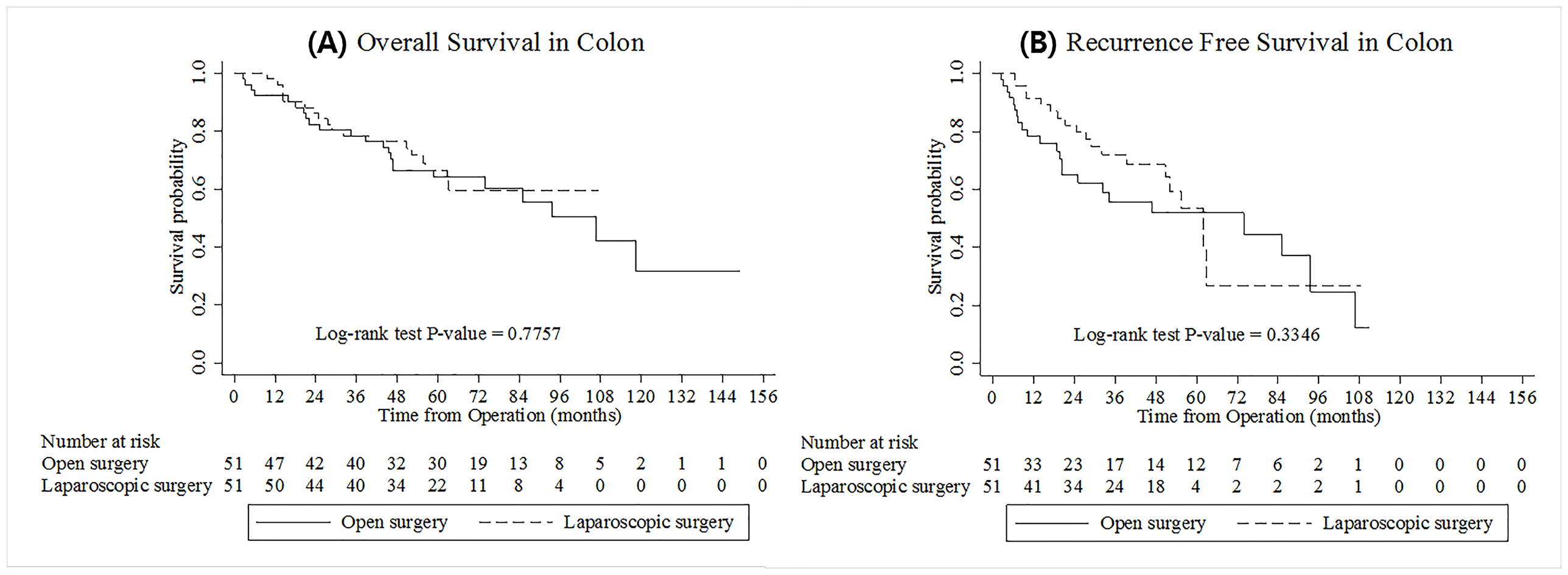

Supplement: Supplementary file 1 — Figure S1. Survival curve in colon cancer patients according to type of surgery. [file CAM4-5-1047-s001.tif]

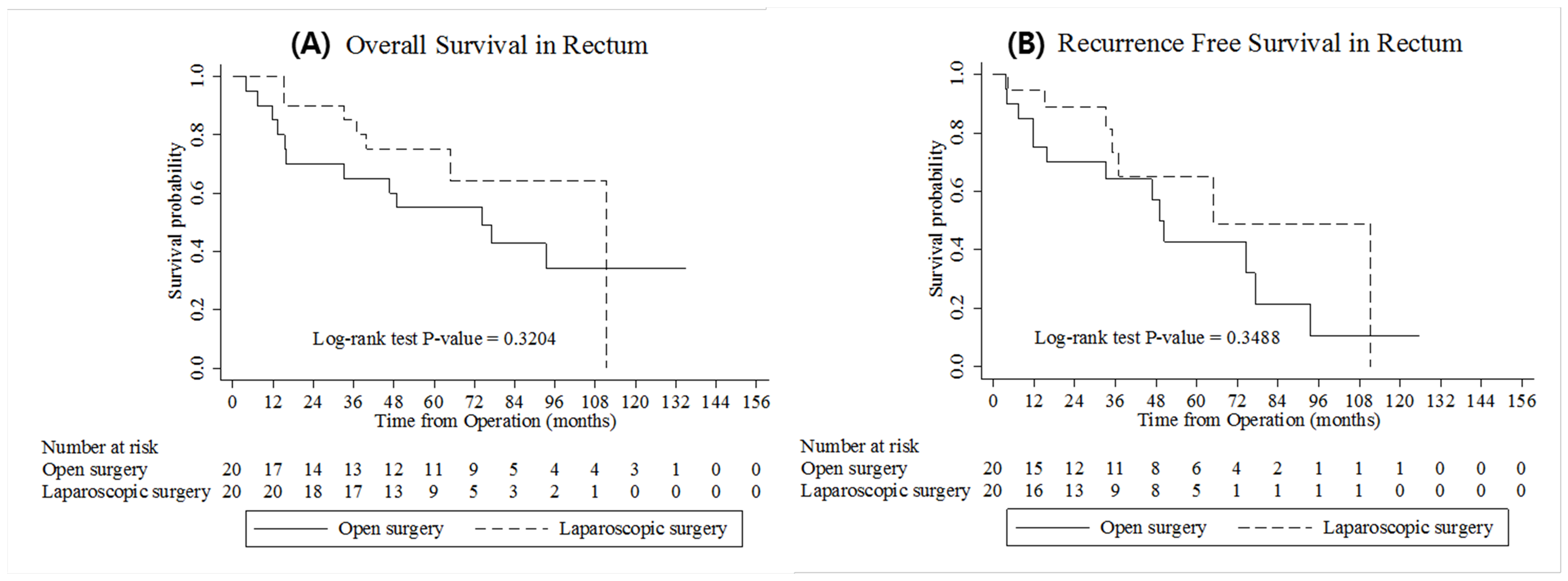

Supplement: Supplementary file 2 — Figure S2. Survival curve in rectal cancer patients according to type of surgery. [file CAM4-5-1047-s002.tif]
