# Supplementary material for: Laparoscopic surgery for patients with colorectal cancer produces better short‐term outcomes with similar survival outcomes in elderly patients compared to open surgery
Source: Cancer Med. 2016 Feb 29;5(6):1047–54. doi: 10.1002/cam4.671 (PMC4924362; doi:10.1002/cam4.671)
Supplement: Supplementary file 3 — Table S1. Factors associated with overall survival in matched cohorts on univariable and multivariable analysis. [file CAM4-5-1047-s003.doc]

Supplemental TABLE 1. Factors associated with OS in matched cohorts on univariable and multivariable analysis

|  |  |  | Univariable analysis | | | Multivariable analysis | | |
| --- | --- | --- | --- | --- | --- | --- | --- | --- |
|  | Alive (n=82) | Death (n=60) | HR | 95% CI | *p* value | HR | 95% CI | *p* value |
| Age (years) |  |  |  |  |  |  |  |  |
| Median (IQR) | 82.0 (81-84) | 82.0 (80.5-83) | 1.02 | 0.91- 1.16 | 0.707 |  |  |  |
| Sex |  |  |  |  |  |  |  |  |
| Male | 39 (49) | 41 (51%) | 1.00 |  |  | 1.00 |  |  |
| Female | 43 (69) | 19 (31%) | 0.59 | 0.34-1.01 | 0.055 | 0.62 | 0.36-1.07 | 0.085 |
| BMI (kg/m^2^) |  |  |  |  |  |  |  |  |
| Median (IQR) | 22.3 (20.8-24.1) | 21.6 (20-25.2) | 0.95 | 0.87-1.02 | 0.172 |  |  |  |
| ASA score |  |  |  |  |  |  |  |  |
| 1 | 14 (50) | 14 (50) | 1.00 |  |  |  |  |  |
| 2 | 52 (63) | 30 (37) | 0.69 | 0.36-1.30 | 0.247 |  |  |  |
| 3 | 16 (50) | 16 (50) | 1.05 | 0.51- 2.16 | 0.888 |  |  |  |
| Comorbidity | 51 (61) | 33 (39) | 0.83 | 0.50-1.39 | 0.481 |  |  |  |
| Hypertension | 47 (64) | 27 (36) | 0.73 | 0.44-1.22 | 0.235 |  |  |  |
| Diabetes mellitus | 9 (45) | 11 (55) | 1.35 | 0.70-2.60 | 0.370 |  |  |  |
| Cardiovascular disease | 3 (43) | 4 (57) | 1.29 | 0.46-3.56 | 0.628 |  |  |  |
| Cerebrovascular disease | 3 (43) | 4 (57) | 1.70 | 0.61-4.72 | 0.307 |  |  |  |
| Pulmonary disease | 1 (50) | 1 (50) | 1.45 | 0.20-10.52 | 0.712 |  |  |  |
| TNM Stage |  |  |  |  |  |  |  |  |
| I | 25 (68) | 12 (32) | 1.00 |  |  |  |  |  |
| II | 26 (54) | 22 (46) | 1.59 | 0.78-3.21 | 0.199 |  |  |  |
| III | 31 (54) | 26 (46) | 1.46 | 0.74-2.89 | 0.278 |  |  |  |
| Tumor location |  |  |  |  |  |  |  |  |
| Right colon | 20 (63) | 12 (38) | 1.00 |  |  |  |  |  |
| Left colon | 41 (59) | 29 (41) | 1.02 | 0.52-1.99 | 0.964 |  |  |  |
| Rectum | 21 (53) | 19 (48) | 1.15 | 0.56-2.38 | 0.703 |  |  |  |
| Preoperative CEA (ng/mL) |  |  |  |  |  |  |  |  |
| ≤5 | 61 (60) | 40 (40) | 1.00 |  |  |  |  |  |
| >5 | 21 (51) | 20 (49) | 1.42 | 0.83-2.43 | 0.205 |  |  |  |
| Type of resection |  |  |  |  |  |  |  |  |
| Right hemicolectomy | 19 (59) | 13 (41) | 1.00 |  |  |  |  |  |
| Left hemicolectomy | 5 (71) | 2 (29) | 0.64 | 0.14-2.85 | 0.559 |  |  |  |
| Anterior resection | 31 (66) | 16 (34) | 0.81 | 0.39-1.69 | 0.577 |  |  |  |
| Low anterior resection | 22 (51) | 21 (49) | 1.08 | 0.54-2.16 | 0.823 |  |  |  |
| Miles’ operation | 2 (50) | 2 (50) | 1.06 | 0.24-4.70 | 0.944 |  |  |  |
| Hartmann’s operation | 1 (20) | 4 (80) | 2.90 | 0.94-8.96 | 0.064 |  |  |  |
| Subtotal colectomy | 2 (50) | 2 (50) | 1.04 | 0.23- 4.60 | 0.963 |  |  |  |
| Operative time (minutes) |  |  |  |  |  |  |  |  |
| ≤ 180 | 55 (63) | 33 (38) | 1.00 |  |  |  |  |  |
| > 180 | 27 (50) | 27 (50) | 1.45 | 0.87-2.41 | 0.153 |  |  |  |
| EBL (mL) |  |  |  |  |  |  |  |  |
| Median (IQR) | 100.0 (50-200) | 100.0 (30-300) | 1.00 | 1.00-1.00 | 0.293 |  |  |  |
| Harvested LN |  |  |  |  |  |  |  |  |
| <12 | 11 (55) | 9 (45) | 1.00 |  |  |  |  |  |
| ≥12 | 71 (58) | 51 (42) | 1.15 | 0.56-2.36 | 0.706 |  |  |  |
| Tumor grade |  |  |  |  |  |  |  |  |
| Low | 73 (57) | 55 (43) | 1.00 |  |  |  |  |  |
| High | 9 (64) | 5 (36) | 0.91 | 0.36-2.27 | 0.834 |  |  |  |
| Venous invasion |  |  |  |  |  |  |  |  |
| No | 61 (60) | 41 (40) | 1.00 |  |  |  |  |  |
| Yes | 21 (53) | 19 (48) | 1.21 | 0.70-2.09 | 0.492 |  |  |  |
| Angiolymphatic Invasion |  |  |  |  |  |  |  |  |
| No | 51 (59) | 36 (41) | 1.00 |  |  |  |  |  |
| Yes | 31 (56) | 24 (44) | 1.25 | 0.74-2.11 | 0.405 |  |  |  |
| Perineural invasion |  |  |  |  |  |  |  |  |
| No | 65 (61) | 41 (39) |  |  |  | 1.00 |  |  |
| Yes | 17 (47) | 19 (53) | 1.94 | 1.11-3.40 | 0.020 | 1.79 | 1.02-3.15 | 0.043 |
| Flatus passage (days) |  |  |  |  |  |  |  |  |
| Median (IQR) | 4.0 (3-5) | 4.0 (3-5) | 0.99 | 0.89-1.09 | 0.771 |  |  |  |
| First soft diet (days) |  |  |  |  |  |  |  |  |
| Median (IQR) | 5.5 (4-7) | 5.0 (4-7) | 0.98 | 0.90-1.07 | 0.695 |  |  |  |
| Hospital stay (days) |  |  |  |  |  |  |  |  |
| Median (IQR) | 9.0 7-11 | 10.0 8-13 | 1.02 | 0.99-1.06 | 0.173 |  |  |  |
| Postoperative morbidity | 23 (51) | 22 (49) | 1.31 | 0.77-2.22 | 0.315 |  |  |  |
| Wound infection | 6 (43) | 8 (57) | 1.61 | 0.76-3.40 | 0.213 |  |  |  |
| Ileus | 9 (64) | 5 (36) | 0.72 | 0.29-1.80 | 0.478 |  |  |  |
| Urinary retention | 10 (56) | 8 (44) | 1.12 | 0.53-2.36 | 0.769 |  |  |  |
| Anastomosis leakage | 0 (0) | 0 (0) | 1.00 |  |  |  |  |  |
| Intra-abdominal bleeding | 1 (50) | 1 (50) | 1.66 | 0.23-12.04 | 0.618 |  |  |  |
| Pneumonia | 1 (100) | 0 (0) | 0.00 |  | 1.000 |  |  |  |
| Other complication | 2 (33) | 4 (67) | 2.25 | 0.81-6.25 | 0.119 |  |  |  |
| Type of surgery |  |  |  |  |  |  |  |  |
| OP | 36 (51) | 35 (49) |  |  |  | 1.00 |  |  |
| LAP | 46 (65) | 25 (35) | 0.81 | 0.48-1.36 | 0.423 | 0.82 | 0.48-1.38 | 0.457 |

Data are presented as n (%) unless otherwise indicated

HR, hazard ratio; CI, confidence interval; IQR, interquartile range; BMI, body mass index; ASA, American Society of Anesthesiologists; TNM, tumor node metastasis; CEA, carcinoembryonic antigen; EBL, estimated blood loss; LN, lymph node; LAP, laparoscopic surgery; OP, open surgery
