# Supplementary material for: Laparoscopic surgery for patients with colorectal cancer produces better short‐term outcomes with similar survival outcomes in elderly patients compared to open surgery
Source: Cancer Med. 2016 Feb 29;5(6):1047–54. doi: 10.1002/cam4.671 (PMC4924362; doi:10.1002/cam4.671)
Supplement: Supplementary file 4 — Table S2. Factors associated with recurrence‐ free survival in matched cohorts on univariable and multivariable analysis. [file CAM4-5-1047-s004.doc]

Supplemental TABLE 2. Factors associated with RFS in matched cohorts on univariable and multivariable analysis

|  |  |  | Univariable analysis | | | Multivariable analysis | | |
| --- | --- | --- | --- | --- | --- | --- | --- | --- |
|  | No recurrence (n=81) | Recurrence (n=61) | HR | 95% CI | *p* value | HR | 95% CI | *p* value |
| Age (years) |  |  |  |  |  |  |  |  |
| Median (IQR) | 82.0 (81-84) | 82.0 (81-83) | 1.01 | 0.89-1.14 | 0.901 |  |  |  |
| Sex |  |  |  |  |  |  |  |  |
| Male | 39 (49) | 41 (51) | 1.00 |  |  |  |  |  |
| Female | 42 (68) | 20 (32) | 0.78 | 0.45-1.35 | 0.382 |  |  |  |
| BMI (kg/m^2^) |  |  |  |  |  |  |  |  |
| Median (IQR) | 22.2 (20.8-24.1) | 21.7 (20-25.1) | 0.95 | 0.88-1.03 | 0.226 |  |  |  |
| ASA score |  |  |  |  |  |  |  |  |
| 1 | 14 (50) | 14 (50) | 1.00 |  |  |  |  |  |
| 2 | 51 (62) | 31 (38) | 0.71 | 0.37-1.34 | 0.289 |  |  |  |
| 3 | 16 (50) | 16 (50) | 0.76 | 0.37-1.56 | 0.453 |  |  |  |
| Comorbidity | 50 (60) | 34 (40) | 0.72 | 0.43-1.19 | 0.196 |  |  |  |
| Hypertension | 46 (62) | 28 (38) | 0.73 | 0.44-1.21 | 0.222 |  |  |  |
| Diabetes mellitus | 9 (45) | 11 (55) | 1.13 | 0.58-2.21 | 0.711 |  |  |  |
| Cardiovascular disease | 3 (43) | 4 (57) | 0.99 | 0.36-2.76 | 0.990 |  |  |  |
| Cerebrovascular disease | 3 (43) | 4 (57) | 1.59 | 0.57-4.43 | 0.371 |  |  |  |
| Pulmonary disease | 1 (50) | 1 (50) | 1.21 | 0.17-8.81 | 0.849 |  |  |  |
| TNM Stage |  |  |  |  |  |  |  |  |
| I | 25 (68) | 12 (32) | 1.00 |  |  |  |  |  |
| II | 25 (52) | 23 (48) | 1.43 | 0.71-2.88 | 0.313 |  |  |  |
| III | 31 (54) | 26 (46) | 1.56 | 0.79-3.11 | 0.203 |  |  |  |
| Tumor location |  |  |  |  |  |  |  |  |
| Right colon | 20 (63) | 12 (38) | 1.00 |  |  |  |  |  |
| Left colon | 41 (59) | 29 (41) | 0.84 | 0.43-1.66 | 0.623 |  |  |  |
| Rectum | 20 (50) | 20 (50) | 0.87 | 0.42-1.80 | 0.705 |  |  |  |
| Preoperative CEA (ng/mL) |  |  |  |  |  |  |  |  |
| ≤5 | 60 (59) | 41 (41) | 1.00 |  |  | 1.00 |  |  |
| >5 | 21 (51) | 20 (49) | 1.84 | 1.07-3.16 | 0.027 | 1.81 | 1.04-3.14 | 0.035 |
| Type of resection |  |  |  |  |  |  |  |  |
| Right hemicolectomy | 19 (59) | 13 (41) | 1.00 |  |  |  |  |  |
| Left hemicolectomy | 5 (71) | 2 (29) | 0.93 | 0.21-4.11 | 0.919 |  |  |  |
| Anterior resection | 31 (66) | 16 (34) | 0.59 | 0.28-1.24 | 0.165 |  |  |  |
| Low anterior resection | 22 (51) | 21 (49) | 0.69 | 0.34-1.41 | 0.312 |  |  |  |
| Miles’ operation | 1 (25) | 3 (75) | 1.33 | 0.38-4.70 | 0.661 |  |  |  |
| Hartmann’s operation | 1 (20) | 4 (80) | 1.03 | 0.32-3.25 | 0.963 |  |  |  |
| Subtotal colectomy | 2 (50) | 2 (50) | 0.56 | 0.13-2.51 | 0.450 |  |  |  |
| Operative time (minutes) |  |  |  |  |  |  |  |  |
| ≤ 180 | 55 (63) | 33 (38) | 1.00 |  |  |  |  |  |
| > 180 | 26 (48) | 28 (52) | 1.24 | 0.74-2.06 | 0.411 |  |  |  |
| EBL (mL) |  |  |  |  |  |  |  |  |
| Median (IQR) | 100.0 (50-200) | 100.0 (30-300) | 1.00 | 1.00-1.00 | 0.169 |  |  |  |
| Harvested LN |  |  |  |  |  |  |  |  |
| <12 | 11 (55) | 9 (45) | 1.00 |  |  |  |  |  |
| ≥12 | 70 (57) | 52 (43) | 1.35 | 0.66-2.79 | 0.412 |  |  |  |
| Tumor grade |  |  |  |  |  |  |  |  |
| Low | 72 (56) | 56 (44) | 1.00 |  |  |  |  |  |
| High | 9 (64) | 5 (36) | 1.58 | 0.63-3.97 | 0.330 |  |  |  |
| Venous invasion |  |  |  |  |  |  |  |  |
| No | 60 (59) | 42 (41) | 1.00 |  |  |  |  |  |
| Yes | 21 (53) | 19 (48) | 1.45 | 0.84-2.51 | 0.180 |  |  |  |
| Angiolymphatic Invasion |  |  |  |  |  |  |  |  |
| No | 51 (59) | 36 (41) | 1.00 |  |  |  |  |  |
| Yes | 30 (55) | 25 (45) | 1.59 | 0.93-2.71 | 0.092 |  |  |  |
| Perineural invasion |  |  |  |  |  |  |  |  |
| No | 64 (60) | 42 (40) | 1.00 |  |  | 1.00 |  |  |
| Yes | 17 (47) | 19 (53) | 2.01 | 1.15-3.50 | 0.014 | 1.90 | 1.08-3.32 | 0.025 |
| Flatus passage (days) |  |  |  |  |  |  |  |  |
| Median (IQR) | 4.0 (3-5) | 4.0 (3-5) | 1.01 | 0.91-1.11 | 0.892 |  |  |  |
| First soft diet (days) |  |  |  |  |  |  |  |  |
| Median (IQR) | 6.0 (4-7) | 5.0 (4-7) | 0.99 | 0.90-1.08 | 0.792 |  |  |  |
| Hospital stay (days) |  |  |  |  |  |  |  |  |
| Median (IQR) | 9.0 (7-11) | 10.0 (8-13) | 1.01 | 0.98-1.05 | 0.516 |  |  |  |
| Postoperative morbidity | 22 (49) | 23 (51) | 1.29 | 0.77-2.17 | 0.331 |  |  |  |
| Wound infection | 6 (43) | 8 (57) | 1.18 | 0.55-2.54 | 0.668 |  |  |  |
| Ileus | 9 (64) | 5 (36) | 0.92 | 0.37-2.31 | 0.860 |  |  |  |
| Urinary retention | 9 (50) | 9 (50) | 1.41 | 0.69-2.87 | 0.346 |  |  |  |
| Anastomosis leakage | 0 (0) | 0 (0) | 1.00 |  |  |  |  |  |
| Intra-abdominal bleeding | 1 (50) | 1 (50) | 1.45 | 0.20-10.57 | 0.714 |  |  |  |
| Pneumonia | 1 (100) | 0 (0) | 0.00 |  | 1.000 |  |  |  |
| Other complication | 2 (33) | 4 (67) | 1.57 | 0.57-4.37 | 0.383 |  |  |  |
| Type of surgery |  |  |  |  |  |  |  |  |
| OP | 35 (49) | 36 (51) | 1.00 |  |  | 1.00 |  |  |
| LAP | 46 (65) | 25 (35) | 0.69 | 0.41-1.16 | 0.162 | 0.67 | 0.40-1.12 | 0.125 |

Data are presented as n (%) unless otherwise indicated

HR, hazard ratio; CI, confidence interval; IQR, interquartile range; BMI, body mass index; ASA, American Society of Anesthesiologists; TNM, tumor node metastasis; CEA, carcinoembryonic antigen; EBL, estimated blood loss; LN, lymph node; LAP, laparoscopic surgery; OP, open surgery
