# Supplementary material for: Laparoscopic surgery for patients with colorectal cancer produces better short‐term outcomes with similar survival outcomes in elderly patients compared to open surgery
Source: Cancer Med. 2016 Feb 29;5(6):1047–54. doi: 10.1002/cam4.671 (PMC4924362; doi:10.1002/cam4.671)
Supplement: Supplementary file 5 — Table S3. Multivariable analysis for overall survival in matched cohorts of laparoscopic and open surgery: subgroup analysis of patients with colon and rectal cancers. [file CAM4-5-1047-s005.doc]

Supplemental TABLE 3. Multivariable analysis for overall survival in matched cohorts of laparoscopic and open surgery: subgroup analysis of patients with colon and rectal cancers

|  | Colon (n=102) | | | Rectum (n=40) | | |
| --- | --- | --- | --- | --- | --- | --- |
|  | HR | 95% CI | *p* | HR | 95% CI | *p* |
| Sex |  |  |  |  |  |  |
| Male | 1.00 |  |  |  |  |  |
| Female | 0.55 | 0.27-1.12 | 0.102 |  |  |  |
| Comorbidity |  |  |  |  |  |  |
| Cerebrovascular disease |  |  |  | 17.00 | 0.70-414.05 | 0.082 |
| TNM Stage |  |  |  |  |  |  |
| I |  |  |  | 1.00 |  |  |
| II |  |  |  | 0.95 | 0.20-4.36 | 0.942 |
| III |  |  |  | 1.71 | 0.38-7.78 | 0.488 |
| Type of resection |  |  |  |  |  |  |
| Low anterior resection |  |  |  | 0.21 | 0.03-1.47 | 0.115 |
| Miles’ operation |  |  |  | 0.17 | 0.02-1.58 | 0.120 |
| Hartmann’s operation |  |  |  | 1.00 |  |  |
| Perineural invasion |  |  |  |  |  |  |
| No | 1.00 |  |  |  |  |  |
| Yes | 1.76 | 0.90-3.45 | 0.099 |  |  |  |
| Postoperative chemotherapy |  |  |  |  |  |  |
| No |  |  |  | 1.00 |  |  |
| Yes |  |  |  | 3.49 | 0.69-17.75 | 0.132 |
| Type of surgery |  |  |  |  |  |  |
| OP | 1.00 |  |  | 1.00 |  |  |
| LAP | 0.94 | 0.50-1.78 | 0.857 | 1.09 | 0.30-3.91 | 0.898 |

TNM, tumor node metastasis; OP, open surgery; LAP, laparoscopic surgery; HR, hazard ratio; CI, confidence interval
