# Supplementary material for: Laparoscopic surgery for patients with colorectal cancer produces better short‐term outcomes with similar survival outcomes in elderly patients compared to open surgery
Source: Cancer Med. 2016 Feb 29;5(6):1047–54. doi: 10.1002/cam4.671 (PMC4924362; doi:10.1002/cam4.671)
Supplement: Supplementary file 6 — Table S4. Multivariable analysis for recurrence‐free survival in matched cohorts of laparoscopic and open surgery: subgroup analysis of patients with colon and rectal cancers. [file CAM4-5-1047-s006.doc]

Supplemental TABLE 4. Multivariable analysis for recurrence-free survival in matched cohorts of laparoscopic and open surgery: subgroup analysis of patients with colon and rectal cancers

|  | Colon (n=102) | | | Rectum (n=40) | | |
| --- | --- | --- | --- | --- | --- | --- |
|  | HR | 95% CI | *p* | HR | 95% CI | *p* |
| Preoperative CEA (ng/mL) |  |  |  |  |  |  |
| ≤5 |  |  |  | 1.00 |  |  |
| >5 |  |  |  | 1.97 | 0.40-9.60 | 0.401 |
| ASA score |  |  |  |  |  |  |
| 1 | 1.00 |  |  |  |  |  |
| 2 | 0.51 | 0.23-1.16 | 0.109 |  |  |  |
| 3 | 0.72 | 0.29-1.80 | 0.483 |  |  |  |
| Comorbidity |  |  |  |  |  |  |
| Cerebrovascular disease |  |  |  | 9.23 | 0.43-197.22 | 0.155 |
| TNM Stage |  |  |  |  |  |  |
| I |  |  |  | 1.00 |  |  |
| II |  |  |  | 0.83 | 0.20-3.41 | 0.798 |
| III |  |  |  | 0.53 | 0.07-3.93 | 0.532 |
| Type of resection |  |  |  |  |  |  |
| Low anterior resection |  |  |  | 0.10 | 0.01-1.40 | 0.087 |
| Miles’ operation |  |  |  | 0.25 | 0.01-4.44 |  |
| Hartmann’s operation |  |  |  | 0.00 |  |  |
| Operative time (minutes) |  |  |  | 1.01 | 1.00-1.02 | 0.136 |
| EBL (mL) |  |  |  | 1.00 | 1.00-1.00 | 0.575 |
| Perineural invasion |  |  |  |  |  |  |
| No | 1.00 |  |  |  |  |  |
| Yes | 2.08 | 1.05-4.14 | 0.037 |  |  |  |
| Postoperative chemotherapy |  |  |  |  |  |  |
| No | 1.00 |  |  | 1.00 |  |  |
| Yes | 2.05 | 1.00-4.17 | 0.049 | 1.85 | 0.30-11.28 | 0.506 |
| Type of surgery |  |  |  |  |  |  |
| OP | 1.00 |  |  | 1.00 |  |  |
| LAP | 0.84 | 0.43-1.64 | 0.611 | 1.05 | 0.28-3.94 | 0.94 |

CEA, carcinoembryonic antigen; ASA, American Society of Anesthesiologists; TNM, tumor node metastasis; EBL, estimated blood loss; OP, open surgery; LAP, laparoscopic surgery; HR, hazard ratio; CI, confidence interval
